# "Research ends with publication": a qualitative study on the use of health policy and systems research in Ethiopia

**DOI:** 10.1186/s12961-023-01091-6

**Published:** 2024-01-02

**Authors:** Sudhakar Morankar, Gelila Abraham, Zubin Shroff, Zewdie Birhanu

**Affiliations:** 1 Ethiopian Evidence Based Healthcare and Development Centre: a JBI Center of Excellence, Jimma University, Jimma, Ethiopia; 2 Health, Behavior, and Society Department, Public Health Faculty, Institute of Health, Jimma University, Jimma, Ethiopia; 3 Health Policy and Management Department, Public Health Faculty, Jimma Institute of Health Sciences, Jimma University, Jimma, Ethiopia; 4 Alliance for Health Policy and Systems Research, World Health Organization, Geneva, Switzerland

**Keywords:** Health system, Health system and policy research, Health research, Evidence-based decision making, Ethiopia

## Abstract

**Background:**

Decision-making about the design and implementation of health care policies should be supported by research evidence. This article reports on a qualitative study on the experiences of both research institutes and policymakers in Ethiopia in generating and using research evidence to inform health policy decision-making.

**Methods:**

Semi-structured interviews were conducted from January through March 2020, with representatives of research institutes and with policymakers in Ethiopia. The data collected during the interviews were analyzed thematically.

**Results:**

Half of the institutions represented had engaged in health policy and systems research (HPSR). These institutes’ capacities were limited by multiple factors, including unsupportive research environments; the limited number of researchers with extensive experience; high turnover among senior researchers; lack of staff motivation mechanisms; underdeveloped research culture; limited technical and analytical capacity among researchers; lack of core funding for HPSR; ineffective financial management; and, lack of connections with health policy platforms. Research institutes also lacked the capacity in strategic packaging of findings to influence policy decision-making, although some programs have recently improved in this area. Meanwhile, there lacked a culture of using evidence in policymaking settings. In general, we found that policymakers had poor attitudes towards the quality or value of the evidence, and had little capacity to interpret evidence and apply findings to policy options. As a result, much of the research produced by the institutes have only been relevant academically, with little impact on policy. However, respondents reported that the environment is slowly changing, and the recent creation of a Research Advisory Council at the Ministry of Health offers a promising model.

**Conclusions:**

Despite some recent changes, in Ethiopia researchers and policymakers alike often tend to consider health policy and systems research (HPSR) to be not very valuable since the findings generated are rarely used for evidence-informed policy development. Research institutes and researchers need to strengthen their technical, analytical, and administrative capacities (through, among other efforts, seeking more funding for research, and better incentives to attract, retain and build skills among qualified researchers); they also need to improve their understanding of the evidence-to-policy cycle and how to engage effectively with policymakers.

## Background

Ethiopia is the second-most populous country in Africa, with a total population of around 110 million, where 80% of the population living in rural areas [1]. In 2017, Ethiopia’s gross domestic product was US$ 772.31 per capita and health expenditure was US$ 66.7 per capita [2] Ethiopia is the Federal Democratic Republic composed of ten regional states. Each regional state is divided into zones that are further divided into districts. The district is a basic decentralized administrative unit with an administrative council composed of elected members [3].

Ethiopia’s current approach to health is based on policies that were initially promoted during a transitional government in 1993. These policies directed more attention to preventive and promotive services [4]. The country has made substantial progress in improving access to health services, particularly since 1997 when it began introducing successive health sector development plans (HSDPs). More recently, the country’s second national Growth and Transformation Plan (GTPII) set ambitious goals to improve quality, equity, coverage, and utilization of essential health services [3]. During the HSTP I, Ethiopia achieved remarkable improvements in several key health indicators. Life expectancy at birth increased from 63 to 66 years by 2018, and access to basic health services increased substantially. In 2019, under-five mortality had reduced from 121 to 55 deaths per 1000 live births and the infant mortality rate declined from 77 to 43 per 1000 live births [5]. Morbidity and mortality from infectious diseases have declined dramatically. However, morbidity and mortality from non-communicable diseases (NCDs), and other behavior-related risk factors are causing growing concern in Ethiopia [3].

**Fig. 1:**
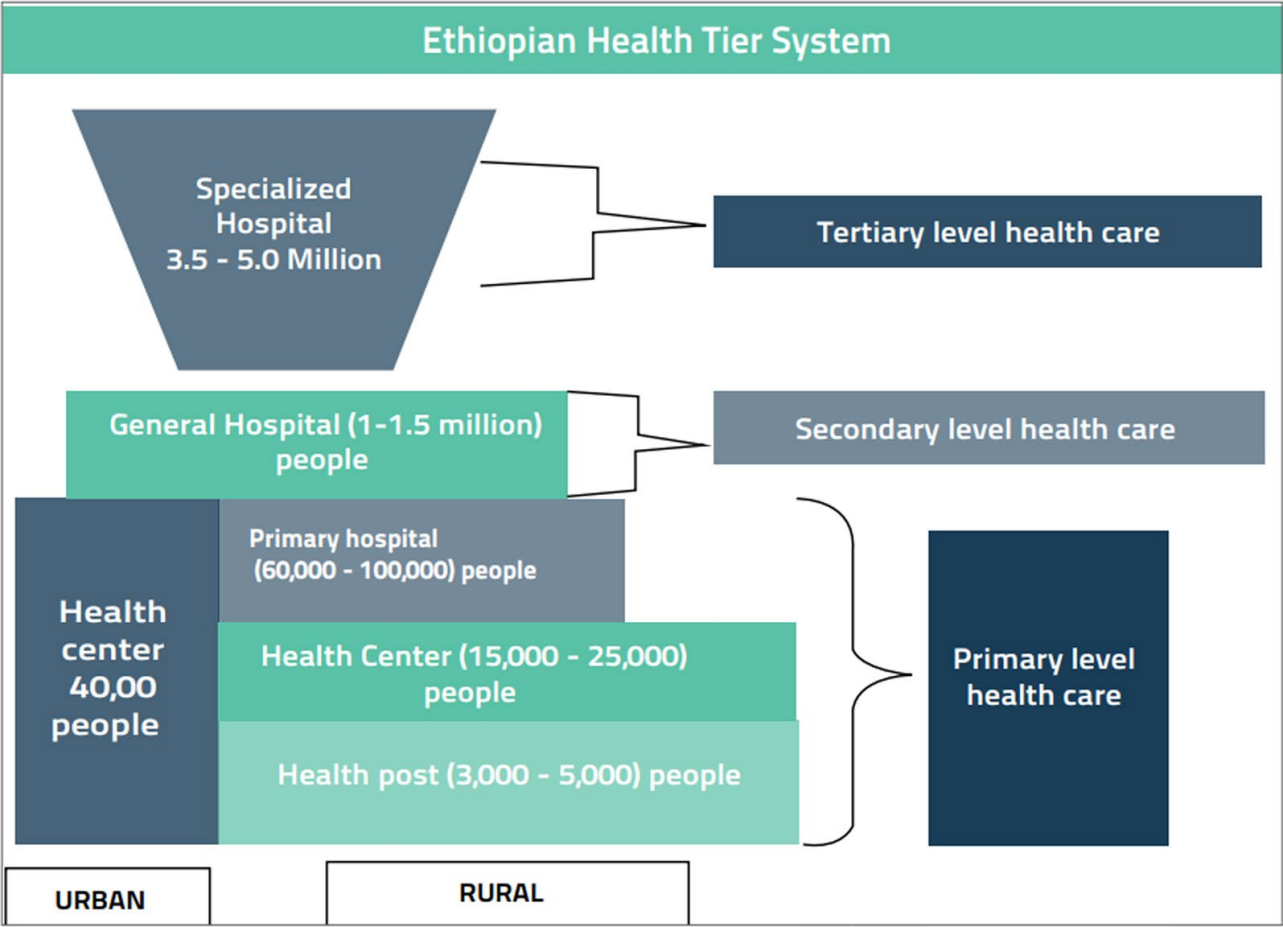
Ethiopian health tier system

The governance of the health system follows the broader context of Ethiopia’s political system [6]: the Federal Ministry of Health (FMOH), in consultation with regional states, develops policies, strategies, guidelines, and standards. However, the regional states are empowered to govern their respective levels of health system and steward resources accordingly [7, 8]. The health care delivery system is organized as a three-tier structure. The tertiary tier is comprised of specialized hospitals, and the second tier is made up of general hospitals. The primary care tier which includes a primary hospital, health centers, and rural health posts, exists at district level [3]. Below this tier is the community-based health care system, the Health Service Extension Program (HSEP) that was launched in 2003 [9]. All hospitals and health centers in the primary tier are managed and directed by their own governing boards which are accountable to regional health bureau (RHBs) or zonal health office (ZHOs), depending on the level of the hospital. Health center management committees are accountable to district administrations [8] (Fig. 1).

Ethiopia’s health research evidence is most often produced by national research institutes affiliated with the FMOH. Historically, academic and research institutes for health began emerging in Ethiopia in the 1950s. The Gondar Public Health College was the first health training institute established, in 1952, followed by the Addis Ababa University Medical Faculty and the Ethiopian Public Health Institute (EPHI) which was established in 1962 and 1995, respectively [10] 11. The Armauer Hansen Research Institute (AHRI) was established in 1970 to focus on clinical and biomedical research [12]. More recently, in 2015 the FMOH established the International Institute of Primary Health Care in Ethiopia (IIPHC-E) [13]. In addition to these government-affiliated research institutes, many academic institutions, such as Ethiopia’s 45 public universities, may also conduct relevant health policy and system research (HPSR) [11].

Evidence-Based Practice (EBP) has increasingly spread across a wide range of policy areas in countries around the globe. However, the extent to which evidence generated through rigorous research is used as technocrats develop and politicians make health policies is largely unknown. This is an especially murky area in low- and middle-income countries (LMICs). Generating the evidence to support EBP requires rigorously implemented, high-quality research and effective translations of evidence into key policy areas [12]. The capacity to conduct HPSR requires institutional arrangements to manage research projects, adequate and sustainable research funding, and the recruitment and retention of a sufficient number of competent and qualified researchers.

In a broader term, HPSR is defined as a type of research that generates evidence on any or several of the six building blocks of the health systems, namely service delivery, information and evidence, medical products and technologies, health workforce, health financing, and leadership and governance [14, 15]. HPSR is dealing with understanding health policy processes and how different actors interact in the policy process, and it focuses primarily upon the more downstream aspects of the health system such as policies, organizations, and programs, but does not address the clinical management of patients or basic scientific research such as cell or molecular structures. The ultimate objective of health systems research is to promote the coverage, quality, efficiency, and equity of health systems [16]. The extent to which evidence generated through HPSR is being used for making policy decisions and in developing guidelines for practice remains largely unknown in Ethiopia. Thus, we designed this study to assess two interconnected topics: the existing capacity among Ethiopian HPSR institutes to generate policy-relevant evidence and communicate about their research; and, Ethiopian policymakers’ interest and experience in using evidence for decision making on health policy. In doing so, we aim for agenda-setting that might help the researchers and policymakers cooperate closely to understand specific HPSR needs, priorities, ensure the relevance of topics, and improve communication, dissemination, and implementation of the research recommendations.

## Methods

### Settings and approaches

The study employed qualitative methods, with data collected from two groups of respondents: (1) HPSR producers and managers (eg. Researchers in national and subnational research institutes, universities, and private research firms), and (2) policymakers (the intended users of HPSR evidence). We collected data from the HPSR producers and HPSR managers, using self-administered questionnaires and semi-structured interviews. The respondents were sampled purposively as follows: First, five HPSR-producing government institutes were identified through a review of organizational documents and literature. We then assessed whether the identified institutes met our eligibility criteria (namely, currently conducting HPSR and/or knowledge translation activities). Eventually, we identified three national government research institutes (GRIs). Seven public universities were also identified that met the inclusion criteria. Finally, two private research firms and five professional associations that met the inclusion criteria were included to understand their roles and experiences in the production of evidence. With policymakers, we used semi-structured interviews with purposively selected respondents considering criteria such as experience and engagement in the policy-making process or program development, and duration of service in the health system.

### Data collection

Following the desk review of institutional publications and websites, four sets of interviews (i.e. (1) semi-structured interviews with researchers and researcher managers, (2) in-depth interviews with selected researchers/managers, (3) structured interviews policymakers, and (4) in-depth interviews with selected policymakers) were conducted to collect data. In the first set, a total of 16 semi-structured interviews were conducted with purposively selected (criteria included level and duration of experience, seniority, and qualifications) researchers or managers from the HPSR-producing institutes. Each respondent completed a questionnaire to provide basic data on the institution (they were informed of the questions in advance to allow them to prepare and organize the needed data through reviewing organization documents and reports). In addition, each respondent was asked six questions regarding the barriers they face in getting evidence to influence policy; their responses for these items were limited to a five-point Likert scale ranging from fully disagrees to fully agree.

In the second set, we then conducted in-depth interviews with fourteen of the 16 representatives of HPSR producing institutes to explore their HPSR capacity, experiences, and challenges. Key informants were purposively selected to represent diverse experiences and a wide scope of HPSR practices. The in-depth interviews were guided by open-ended questions on organizational experience, capacity, culture, and demand concerning HPSR.

The third set of interviews focused on policymakers as the intended users of HPSR. The purpose of these interviews was to explore the policy environment for HPSR, such as the demand for and supply of evidence, and the culture and trends of evidence uptake for health policy-making in Ethiopia. A total of 24 evidence users from different levels of the health system (the FMOH, RHBs, and key FMOH partners) were asked to respond to a seven short written structured questionnaire on the extent of evidence uptake for policy development. Each of the seven questions used a four-point Likert scale (from “strongly disagree” to “strongly agree). In the fourth set, eleven of the 24 respondents were then purposively sampled (based on their level of experience) to participate in an in-depth interview on the culture of evidence use for health policy in Ethiopia.

To help the respondents distinguish HPSR from other health researches, a brief explanation was given to each respondent both in written and explained by an interviewer. The in-depth interviews were conducted by one or two senior members of the study team in either English or Amharic, depending on the preference of the respondent. Each in-depth interview was recorded using a digital voice recorder, and interviewers also took notes. Interviews ranged from 35 to 63 min in duration. All interviews were conducted either at respondent’s office or a separate discussion room (based on their preference). Informed verbal consent was taken from all respondents at the beginning of the interview.

### Data analysis

Data analysis procedure was performed according to existing analysis framework [17, 18]. First, the recordings of the in-depth interviews were transcribed verbatim and translated into the English language by the study team. The analytic method was thematic analysis through an open coding technique, according to Mattew B. Miles and A. Michanel Hberman and Elizabeth et al. [17, 18]. Coding and analysis of the transcripts were assisted by ATLAS.ti 7.1.4. ZB and GA, independently read, re-read and coded selected transcripts to come up with an initial coding structure through iterative rounds of open coding of emergent themes associated with the research objective and guided by grounded theory [19]. To ensure the relevance and appropriateness of the coding structure, another investigator (MS) reviewed and verified the code structure. All the transcripts were then coded by ZB. After all the transcripts were fully coded, the investigators (lead by ZB), organized the codes in a systematic order and categorized them based on shared characteristics. All authors discussed and standardized the emergent categories which led to themes and subthemes. Finally, the results were organized by subthemes and elaborated with selected quotes. The quantitative data were analyzed using SPSS Statistics 21.0 using pre-defined categories. The data from the qualitative in-depth interviews were triangulated with the data from the self-administered questionnaires. The consolidated criteria for reporting qualitative research (COREQ) were followed to report the data [20]. The findings were presented to stakeholders and feedbacks were obtained from participants.

## Results

### Profile of respondents

Table 1 shows the profile of the study participants. In total, 40 researchers and policymakers participated, of whom 16 respondents were from research-producing institutes, and 24 were policymakers at two levels (Table 1). All respondents were male except one female. Eight respondents had Ph.D. and the remaining had masters’ degrees.

**Table 1:** Profile of respondents included in the study

Characteristics	Categories	*N* (%)
Respondent categories (*N* = 40)	HPSR producers	16 (40.0)
	Policymakers	24 (60.0)
Policymaker decision level (*N* = 24)	National	16 (66.7)
	Regional	8 (33.3)
HPSR organization types (*N* = 16)	University/academic institutes	7 (43.8)
	Research institutes	3 (18.7)
	Private research firms	2 (12.5)
	Professional Associations	4 (25.0)
Years of experience (HPSR producers *N* = 16)	< 5 years	5 (31.2)
	> = 5 years	11 (68.8)

The study findings are organized into three broad themes: the capacity for conducting HPSR in the country, the research environment, and the uptake of evidence in the development of policy.

### Capacities of HPSR producers

Only approximately half of the organizations included in the assessment reported conducting health policy-related research; other health research activities focused on biomedical and clinical research. Two GRIs recently established a separate department for health system research and knowledge translation activities.

#### Functions and roles of research institutes

All of the research-producing organizations reported offering training, workshops, and short courses to diverse audiences including postgraduate students, researchers at different levels, and sometimes experts from the health system. Almost all of the organizations also said they conduct policy and advocacy to influence decision-makers. Other functions of the institutions included offering degree programs, monitoring and evaluation of programs, and allocating funds to other organizations. Universities conducting clinical or biomedical research reported disseminating their findings mostly through publications and annual research conferences; they rarely produced policy briefs. They did report engaging in collaborative research with ministries and other stakeholders and participating in consultative workshops and meetings at regional or national levels. Private institutions also reported conducting training for the health workforce in partnership with public universities. Private institutes reported mostly conducting research based on contracts or commissions from ministries; they typically share and disseminate reports on their findings, but rarely produce policy briefs and recommendations. One respondent stated:

*We conduct two types of research: small-scale, funded by [our] organization, and large-scale with a huge amount of funding from a client—FMOH or an external funder—to evaluate programs nationwide, e.g. family planning and decentralization of health services at the community level. (KII, Private Research Institute)*

Professional associations conduct research mostly in collaboration with academic institutions, FMOH, or other partners. These institutions sometimes do produce policy recommendations and briefs and undertake advocacy and communications to the FMOH to influence evidence uptake. They also support FMOH on the development of national health programs, policies, guidelines, and standards in their respective professional categories; hold conferences and workshops; conduct capacity building on research skills (including grant writing); and implement projects in collaborations with stakeholders. Three of the professional associations publish scientific journals and three provide ethical reviews and clearance letters to researchers. According to some interviewees, the FMOH trusts professional associations, which enables them to develop strong collaborations and relationships. One professional association respondent stated:

*FMOH trusts us; we involve them in the majority of our activities and intervention, provide on job mentorship. We participate in the training and supervision of health services. FMOH considers us as one of its departments in the ministry and as a supporting partner. (KII, Professional Association)*

#### Human resources for HPSR

Table 2 profiles the researchers working in research institutes and universities. The human resources situation available to HPSR producers is characterized by inadequate staff; further, they have few senior and experienced researchers, with the majority of the institutes’ staff members are early in their careers. The largest proportion of available staff with a wide variety of expertise is found in universities, followed by public research institutes. Senior researchers are concentrated in two universities, and 240 senior researchers are based in a single institution. Likewise, a larger number (87) of experts with an educational level above a master’s degree were found in universities as compared to other types of research institutes. However, professional associations and private research institutes reported that they did not have adequate permanent/regular research staff.

**Table 2:** Number of research staff at institutes, February 2020

Staff	Type of institution				
	University (*N* = 7)	Public Research Institute (*N* = 3)	Private Research Institute (*N* = 2)	Professional Associations (*N* = 4)	Total (*N* = 16)
	Average (Min, Max)	Average (Min, Max)	Average (Min, Max)	Average (Min, Max)	Average (Min, Max)
*Academic rank*					
Senior Researcher	52 (10, 240)	34 (1, 91)	9 (3, 15)	0.8 (1, 2)	30.5 (1, 240)
Researcher	92 (30, 312)	59 (0, 165)	6.5 (3, 10)	0.8 (0, 3)	52 (0, 312)
Research Assistant	33 (0, 87)	49 (0, 121)	16.5 (3, 30)	0 (0, 0)	26.8 (0, 121)
*Qualifications*					
Higher than master’s	28 (3, 87)	27 (1, 67)	4.5 (4, 5)	0 (0, 0)	18 (0, 87)
Master’s degree	96 (30, 312)	118 (0, 203)	12.5 (5, 20)	1 (0, 4)	66 (0, 203)
Bachelor degree	32 (0, 162)	87 (0, 157)	0 (0, 0)	0.3 (0, 1)	30 (0, 162)

All categories of respondents in the in-depth interviews consistently explained that insufficient experienced and qualified experts were available to meet demand in the market; in particular, lack of retention and motivation mechanisms affected the availability of qualified research staff in research institutes. Unattractive salaries, absence of staff tracking, and poor staffing plans contributed to poor human resource development, as elaborated in Table 3. Universities and other institutions described offering capacity-building opportunities to staff, including short courses, training, and workshops, mentoring internships, or fellowship programs. However, KIs consistently reported that these opportunities were not sufficient to effectively build the capacity of researchers.

**Table 3:** Challenges cited related to poor human resource capacity in research institutes

Category	Elaborative quote
Lack of skilled, qualified, senior, or experienced researchers	Poor response to our vacancy announcements. We do not receive enough applications with the required qualifications. We recruit people who have less skill and work experience. We have to train them, and after getting the training and acquiring enough experience of one or years, they leave us. That’s why we say it seems [we are a] training center. (KII, Research Institute)
Unattractive salaries, frequent staff turnover, and inadequate staffing level	In public sector health institutions, the salary for researchers may be very low. So, it will be very difficult to retain and motivate high-caliber researchers in the field; so this is also a very important part in which the civil service and the government should also think of arrangements for these calibers. (KII, National Policymaker)
Weak research culture among researchers	Research cultures (i.e. in terms of ethics, transparency, practical based, multidisciplinary approaches, creating synergy, designed system, terminal report regardless of who funded it, etc.) are not deep-rooted in each of the staff members. Among all these reports, project management is the main weakness (KII, University)
Poor/lack of a plan for human resource and retention of experienced and skilled researchers	You find a human resource component is still a pressing challenge because keeping an experienced researcher in place is difficult. The retention issue we have, [there have been] some improvements currently but when the market outside the institute is lucrative, researchers go away. (KII, Research Institute)

#### Availability of HPSR funding

Key informants indicated that no health research institute had a separate core budget for HPSR. Instead, HPSR was considered to be an integral part of health research. Public research institutes received funding from the government through FMOH, and from international sources, while universities research funds were allocated to them by ministry of science and higher education (MOSHE). Five of 11 (45.5%) of the institutes reported that they obtained research funding exclusively from the government, while the remaining institutes receive funding from both government and non-government sources. Seven of the 11 institutes (63.6%) said the source of funding for their HPSR was international. Across all research institutions, most funds were obtained through competitive grant applications and collaborations at national and international levels. Key informants across institutes stated that the government allocated only a small amount of funding for health research. Universities in particular argued that the lack of adequate funds from the government is a critical bottleneck to the generation of useful evidence. Further, inadequate funds limit the institutes’ ability to engage relevant stakeholders in the research process. One university-based interviewee said:

*There is a wide gap between the contextualized problems and researches being conducted by our researchers. This was due to the amount of budget allocated by the government for universities, [which] is almost nil. (KII, University)*

Another challenge facing researchers seeking to do HPSR is inappropriate arrangements for and use of the available funds. This challenge comprises several factors, including excessive bureaucracy, lack of transparency, short funding periods, and poor utilization of funds at institutional levels. These were reported by interviewees as creating additional barriers to health research in universities. Except in a few instances, universities reportedly do not have departments or units dedicated to the management of earmarked research funds; rather, research fund management is treated as part of the routine management of government-allocated resources. Some universities reportedly do not apply the percentage of external funding allocated to overhead and administration to improve research processes, as one respondent said:

*The projects are independent, but the salary is paid by the government budget. I do not know why the project individuals do not recruit their own professionals working on finance, why the overhead amount is not pooled, and why finance is not managed by strong professionals. (KII, University)*

A GRI representative also reported receiving inadequate funds to conduct high-quality HPSR:

*The quality, effectiveness and operational aspects of health system research did not mature enough due to limitations in a financial capacity. (KII, GRI)*

Private research institutes can only get access to government funds through bidding on calls for proposals issued by the FMOH or RHBs. A few of them occasionally receive research grants (either directly or via the government) from bilateral or multilateral partners, such as USAID, WHO, and UNICEF, to provide support to government programs. However, these grants are often insufficient to cover the full costs of high-quality research. Further, they only support specific projects, leaving the institutions without sufficient financing to produce high-quality research evidence on topics outside program areas selected by donors. As one interviewee stated:

*Unless we have really good financial support, it is difficult to do quality research. So financing, capacity, and relationships with the health policy decision-makers are areas to be improved in private institutions. (KII, Private Research Institute)*

The professional associations rarely initiated HPRS as they do not have their budgets for research activities. However, they collaborate with the MOH, RHBs, universities, and other partners in conducting research. A few professional associations have responded to calls for proposals or received direct invitations from the government or NGOs to conduct research. One described:

*We should underscore that lack of funds substantially limited our research activities; our funding mainly comes from international donors. (KII, Professional Association)*

There are in fact strict limits to the amount of international funding that any NGO in Ethiopia can access. The Ethiopian government’s charities and societies proclamation [21] restricts organizations from having more than 10% of the total organizational budget provided by foreign funders. Finally, across the board, respondents noted that lack of research funds affects institutions’ ability to retain experienced researchers.

### Context for using HSPR to develop health policy

Despite numerous challenges with producing HPSR, the environment is increasingly positive and encouraging regarding the uptake of research evidence for policy and decision making. For example, the FMOH has established a Research Advisory Council (RAC) in the Maternal and Child Health (MCH) directorate. The RAC serves as a platform to promote evidence synthesis and uptake, create demands for evidence, and link research producers with policymakers. The country’s most recent Health Systems Transformation Plan (HSTP-II) [3] which placed particular emphasis on “the information revolution”, was mentioned as fostering a positive environment for evidence-based health care policy. Key informants acknowledged that these recent efforts were designed to address the challenges noted above by creating a more supportive environment for HSPR to grow, although the efforts were still in their infancy stage with ongoing challenges. Currently, the environment still often makes it difficult for researchers to both conduct HPSR and to see results appreciated and applied. One researcher working at a university said:

*The existing environment was entirely unsupportive to researchers: limited government funding; absence of capacity building, lack of learning opportunities, and poor mentorship in research; lack of robust grant management system resulted in bureaucratic procurement and financial processes; poor transparency; poor or absent of incentives and guidance to researchers; lack of awareness about research integrity due to lack of rules governing research practices; lack of research facilities and procedures to support researchers. Different departments and research centers within and between universities lack an effective and sustainable system of sharing available scarce research resources and facilities. (KII, University)*

Five of the universities included in the assessment have their own scientific journals, which contributes to research dissemination. Full access to domestic peer-reviewed journals was available at three-quarters of the institutes, but less than half have full access to international peer-reviewed journals (42.9%) and statistical databases (35.7%). Universities and research institutes have more access to these supportive facilities than professional associations. University research centers on various health themes (e.g. tropical and infectious diseases, microbiology, bacteriology, and drug quality assurance), as well as established sites for field research and demographic and health surveillance, were also recognized as contributing to the enabling environment for HPSR.

Overall, we found a lack of specific policy guidance regarding the roles and responsibilities of researchers and research institutes for the generation and uptake of research evidence. This gap undermines HSPR production and utilization for policy. One key informant stated:

*It is important to redefine the roles and responsibilities of research evidence suppliers and demanders [in order] to bind evidence production to utilization. (KII, partner).*

#### Mechanisms to prioritize HPSR topics

**Fig. 2:**
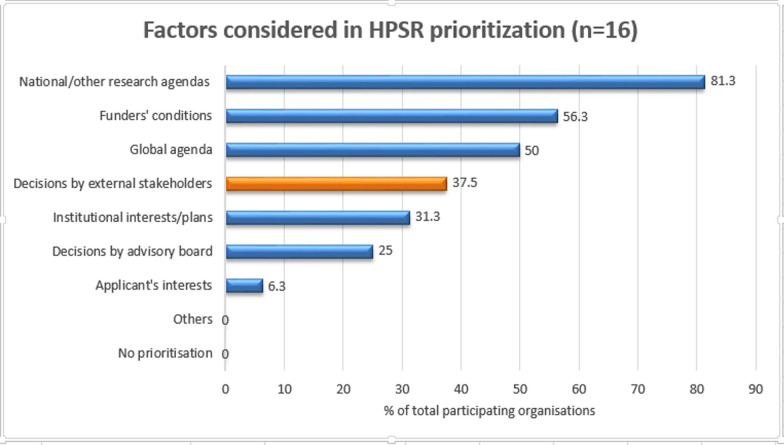
Factors considered in HPSR prioritization engagement among evidence producer organizations in Ethiopia, 2020

Interviewees from research institutes were asked which topics they prioritized for HPSR, and how they arrived at their rankings. Figure 2 shows the factors that respondents reported play into prioritizing areas for HPSR. National priorities and funders’ conditions were common factors. Half of the institutes reported that they were influenced by the global HPSR agenda. The remaining factors were institutional interest and plans, and decisions from advisory boards, committees, or other internal structures. However, apart from following general thematic areas set nationally, few mechanisms exist either to systematically prioritize research areas or to engage with relevant stakeholders.

GRIs frames their thematic research areas based on their mandates—they have their own general research priorities but none specify HPSR. However, key informants from these institutions mentioned that they are well-positioned to understand the priorities of the government. None of the universities had clear HPSR prioritization mechanisms, nor did they systematically engage relevant stakeholders in the generation of evidence. While some key informants mentioned that their research addressed national and international priorities, only one university mentioned consulting with the RHB when establishing research priorities. However, a key informant reflected that this did not necessarily translate into the use of the evidence they generated:

*We try to connect all our research activities in collaboration with the RHB, but could not influence the policy and health system. (KII, University)*

#### HPSR activities, themes, outputs, and quality

Table 4 presents the average number of activities conducted by those institutions that reported HPSR activities. The average annual number of HPSR projects (from 2016 to 2018) was 12 or 13 across all institutes, but with a wide range. The top five themes of the HPSR conducted by the participating organizations in the past three years were: maternal and child health, reproductive health, service delivery, communicable diseases, and non-communicable diseases. During the in-depth interview, respondents also indicated that health human resources, road traffic and injuries, quality of services, and decentralization were also themes addressed in HPSR.

**Table 4:** Research collaboration (within and outside the country) among evidence producer organizations in Ethiopia, 2019 (*n* = 13)

Research collaborations	Within country		Outside country	
	No. of organization	Percentage	No. of organizations	Percentage
Jointly conduct research	13/13	100.0	12/13	92.0
Jointly author publications	11/13	84.6	11/13	84.6
Jointly provide capacity building	10/13	76.9	11/13	84.6
Jointly advocate policy	6/13	46.2	1/13	7.7
No collaboration	0/13	0.0	1/13	7.7
Others	1/13	7.7	0/13	0.0

In our survey, we found that the average number of HPSR related peer-reviewed articles per institution ranged from 48 to 53 between 2016 and 2018; more were published in international peer-reviewed journals than domestic publications. This average masks wide variation among the surveyed institutions: the majority of publications were produced by six groups: two public research institutes and four universities, while the remainder did not publish at all on HPSR topics. Universities, research institutes, and some professional institutes also produced other research outputs, such as reports, policy briefs, books, opinion pieces, presentations in national and international conferences, and public events.

In terms of the perceived quality of research outputs, researchers felt they performed well, but most policymakers contested this assessment. Across five dimensions of quality (timeliness, policy relevance, feasibility, deliverables, and completeness) research organizations scored themselves an average of 4, on a scale of 1 (poor) to 5 (excellent). A large proportion also reported prioritizing their activities based on national or sub-national research agendas, although over half said they considered global priorities, and the same proportion were guided by funding conditions. However, none of the participating organizations could clearly articulate how prioritization happens in practice, for example, through formal mechanisms for selecting research themes, involving key stakeholders. Policymakers were not at all positive about the quality of locally generated research, with many reporting that studies lack quality and actionability.

Research quality assurance methods were used by most institutions; internal peer review was the most widely used (91.7%), followed by external peer review (83.3%) and consultative meetings (75%). Despite these measures, policymakers expressed concern about the quality and relevance of research outputs and partners noted that decision-makers did not have confidence in the findings. As one interviewee said:

*There is enough research. I don’t think the number is an issue; of course, there is always room for change. First, we need to look at the quality of the research outputs. Nowadays everyone is complaining that the research which is done is not proper or not of good quality. This is because we have a very tiny area; we don’t involve experts from other fields; we don’t do large-scale research (projects). (KII, partner)*

### Factors influencing uptake of HPSR evidence

Even when institutions produce research on relevant topics, use quality assurance measures, and disseminates their results; their findings are rarely accepted for use by policymakers. All key informants, including researchers and policymakers alike, consistently reported that HPSR represents a waste of resources because it is rarely used for policy decisions.

The failure to use HPSR evidence also seems to stem from a lack of demand on the part of the policymakers. Several key informants argued that policymakers did not have a good appetite for evidence. Instead, they tended to rely on experts’ opinions, their own “common sense,” “intuition”, or “experience,” or to make politically motivated decisions. A senior researcher described the typical approach: “Usually they establish technical advisory groups or committees of experts and get advice. They outsource the research question and get some documents and [then] mostly align with political direction.” Respondents further suggested that at regional and lower levels of the health care system HPSR is not used at all for health system policy and decision making.

An exception at the federal level was noted. Respondents cited recent efforts and a positive attitude regarding evidence use among program personnel and decision-makers: *“The MCH directorate—it has [a] unique appetite for evidence use. I think this is due to the existence of research advisory council (RAC) for this directorate.” (KII, MOH Policymaker).*

### Mechanisms for influencing policy

**Fig. 3:**
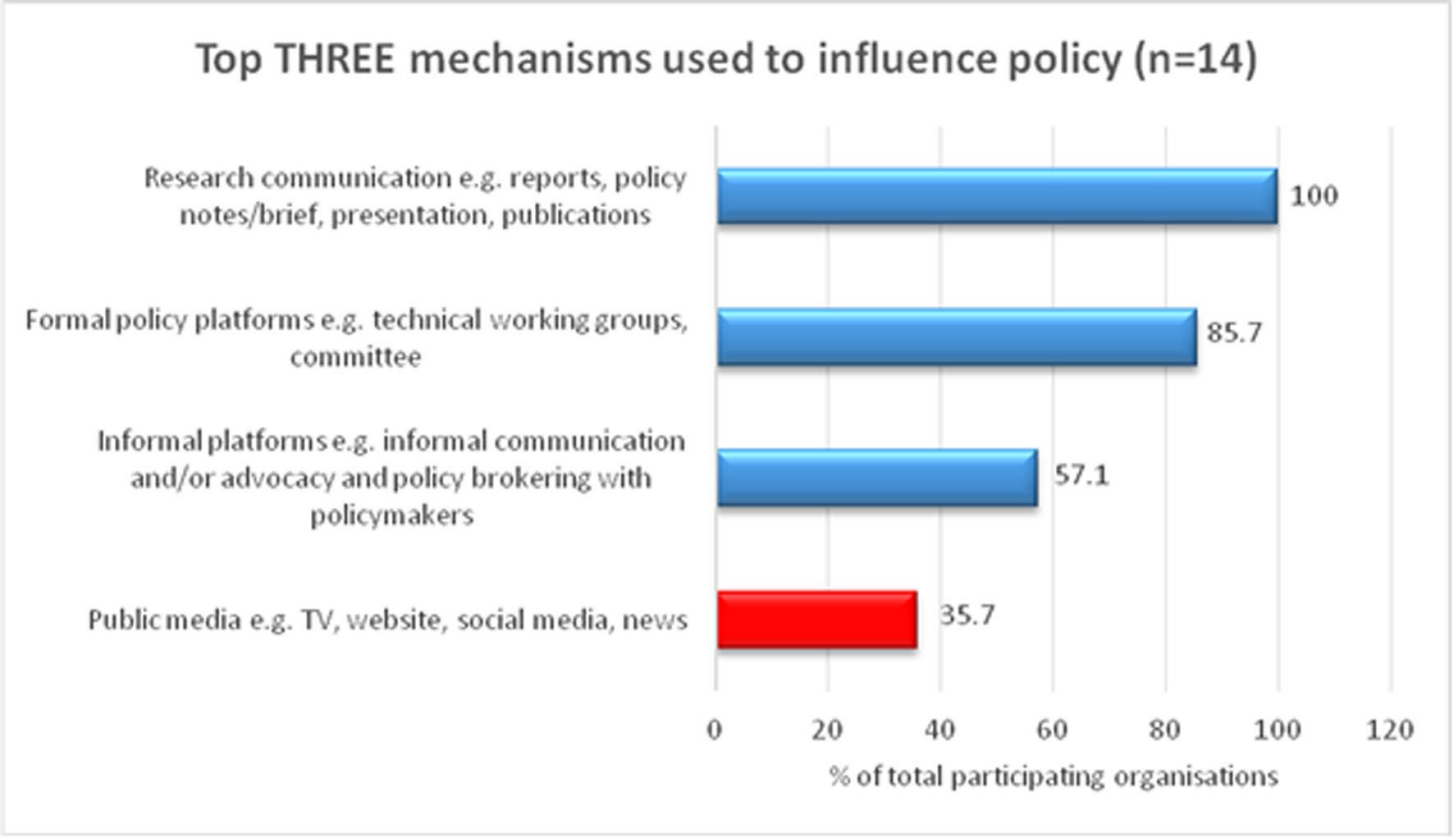
Top three mechanisms used to influence policy by evidence producing organizations in Ethiopia, 2019 (*n* = 14)

Despite the perceived lack of use of HPSR evidence, the research institutions do try to convey their findings. Communication about research results—via reports, policy briefs and notes, presentations, and other publications—was the main mechanism to influence policy mentioned by all surveyed organizations. Formal policy platforms (e.g., technical working groups or committees) and informal policy platforms (e.g., informal communication, advocacy, or other brokering with policymakers) were the second-and third-most mentioned mechanisms. Public media, such as television, websites, social media, and news reporting, was the least used mechanism by the organizations (Fig. 3).

Of the research institutions included in the assessment, 80% reported that they had strong linkages with policymakers. GRIs, which have a mandate to support MOH, reported sharing their research outputs with MOH and other relevant stakeholders in various formats, including technical summaries, abstracts, full reports, policy briefs, and policy dialogues. Private research firms and other professional organizations reported conducting research based on requests from stakeholders and Ministry officials. As a result, these agencies were more likely to communicate about the evidence they generated, and thus to influence policy, including through face-to-face discussions with the MOH or other partners.

However, our qualitative findings indicated that most university-based research institutes had either weak or no links with policymakers. Indeed, key informants reported that no mechanisms or systems exist to link research institutes with policymakers to enable evidence to inform policy. Instead, research institutions were producing evidence to generate professional publications but had no systematic approach to communicating their findings to policy-makers. One informant reported,

*“GRIs and universities do not share their research with policy and decisions makers. A majority of researches end with publications, where no one knows where it goes after that”* (KII, Partner).

Universities tended to share research findings through publications and by organizing annual research dissemination conferences, for which they produce abstract books and conference proceedings. Universities do invite policymakers to attend the conferences; however, the conference format is unlikely to appeal to policymakers and their engagement typically only involves making remarks at the opening or closing ceremonies.

#### Trends in demand for HPSR

Key informants from various categories agreed that the culture of demand for and use of HPSR was currently low or non-existent at all tiers of the health care system. Some informants underscored that use of evidence was particularly absent in decision-making processes at mid-and lower-levels of the health system. Key informants used expressive terms to describe the current lack of demand for and use of evidence for policy, including: “big problem,” “far behind,” “immature,” “limited,” “poor,” and “weak.” One policymaker echoed these opinions: *“There is no strong culture of utilizing the service report, research outputs and any form of evidence to guide the policy, supportive supervision and policy recommendations”* (KII, Policymaker).

Despite this situation, many informants reported that the demand for HPSR is actually improving. Eleven HPSR institutes reported an increasing trend in demand from policymakers for HPSR and ten reported improvements in the culture of evidence-based decision making. Some MOH initiatives are becoming “facilitating factors” for encouraging a culture of evidence use, such as the establishment of the RAC for maternal, nutrition, and child health, engagements with international and local universities, using technical working groups to develop and revise guidelines, allocating small budgets to evidence-uptake efforts, and the government’s effort to promote an “information revolution.” A development partner noted that changes are happening:

*“Yes, there is a positive change: a positive attitude toward evidence use among policymakers. There are certain initiatives that MOH has to use evidence, to establish the structure. Still, they are trying how to get the best way”* (KII, partner).

#### Barriers that prevent use of evidence for policy

**Fig. 4:**
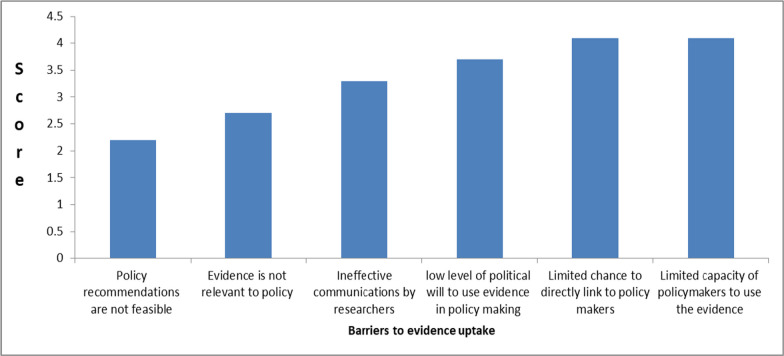
Barriers in getting evidence to policy rated by evidence producing organizations in Ethiopia, 2019 (*n* = 13)

According to the research producers, the two main barriers to the use of the evidence for policymaking were: limited capacity among policymakers to use evidence (see Fig. 4); and, lack of channels to link with policymakers. Other factors mentioned included: low level of political will to use evidence in policymaking; ineffective communication by researchers; and the relevance and feasibility of the evidence produced.

Policymakers and partners also identified several barriers to the use of evidence for policy, as described below with illustrative quotes. The barriers are presented in order of most to least frequently mentioned. Unlike the research producers, policymakers were not especially concerned about their capacity to understand or utilize the data. Instead, they most often cited the failure of the researchers to effectively disseminate and communicate their findings as a barrier. They also noted that because policymakers are not involved in the research, and due to concerns about the quality and relevance of research, the evidence generated is not of interest or useful to them.

### Ineffective dissemination and communication

We don’t really get timely information or evidence. We just act without evidence. For instance, in the case of [the] measles outbreak, we don’t know why it occurred and what was wrong in the system.. We just try to manage the outbreak without understanding the factors that contributed to the ongoing outbreak. We don’t [have] timely access to information or evidence; we are in search of evidence but we don’t get it. (KII, Regional policymaker).

### Disconnection and absence of engagement of policymakers

Policymakers are not part of the research from the beginning. No research institutions engage policymakers from the beginning of their research processes. For instance, if you take the practice of [research institution], they would conduct the study or the survey, write down reports and send the report to the Ministry, and finally may conduct a dissemination workshop. That is all their efforts! But this can never bring utilization of evidence. [KII, National Policymaker].

### HPSR culture and trend

There are gaps in the use of the evidence that is generated. There is no strong culture of utilizing the service report, research outputs [or] any form of evidence to guide the policy, supportive supervision [or] policy recommendations. This poor utilization of evidence even [gets worse] when you go down to districts. (KII, National Policymaker).

### Lack of motivation, attitudinal and value for evidence

The problem is that program personnel often do not pay attention to research findings. They usually consider research findings that are generated after many ups and down as useless. This is due to a lack of awareness about the importance of evidence in program implementation. They don’t give value to evidence! Rather they would give more attention to word of mouth from the ministry than research evidence. (KII, Regional policy maker).

### Irrelevant and poor quality research

Universities are among the major research institutes in the country; the problem is that they focus more on theory and they don’t know the current situation, our strategies. So they conduct research which is in the air, a vacuum. So we are not using it. (KII, National Policymaker).

### Lack of demand, access, and evidence selection

I observed that most of the offices are heavily immersed in day-to-day routine activities. They don’t have even time to read in detail. So they need much briefer policy documents that can guide them in their activity. Whenever you start to work with them, sometimes they cannot continue with you because they are hugely involved in meetings, workshops, travels, and so on. They don’t have time even to browse evidence. So important key points and well-articulated evidence presented to their table are quite paramount. (KII, National Policymaker).

### Lack of evidence translation system, platform, and leadership

There is no accountability. In our review meetings here in the Bureau and the Federal Ministry of Health, research institutes are not evaluated for their performance. How many research projects are done, what does the evidence inform? [It] is not clear to me. There is no mechanism of accountability to uptake the evidence. Sometimes what research questions are done in the research institutes, whether it is in the health sector or the agriculture sector, is not documented. This poses a challenge to the uptake of the evidence. (KII, Regional Policymaker).

### Lack of incentives or supportive environment

The absence of an incentive package and lack of rewarding high impact policy change publications are some of the problems which made the staff focus only on publication and made the studies not influence policies. Our staff could do more influential researches than this—if the recognition or rewarding systems are improved. (KII, University).

### Budget limitations

Unless we have really good financial support, it is difficult to do quality research. Doing quality research all depends on adequate funds. So financing, capacity, and relationships with the health policymakers or decision-makers are areas to be improved in private institutions. (KII, Private Research Institute).

### Lack of capacity and expertise by policymakers

There is a shortage of human power, as we have many different case teams and there is not enough human power that works on policy analysis and [similar] activities. So policy development was not enough. (KII, National Policymaker).

### Political motivations and external influence/environment

Decisions are made politically: someone is the boss, they make the decision, and people will follow. And this specifically has to do with, for example, how the health extension workers doing things. So, someone started it some years back, and there have been many studies about them, but do you see anything changing? No, nothing is changing! Whenever someone in the ministry wants to do some policy, there are policy moments (KII, National Policymaker).

## Discussion

This study assessed whether health policy and systems research in Ethiopia is conducted and used for decision-making by policymakers. The findings are concerning on two levels: HPSR capacities and research uptake.

### HPSR capacities

#### Less qualified human resources

Little HPSR is being conducted by Ethiopian research institutes due to several reasons, and the first is their limited capability.; HPSR is not incorporated as a central activity in many domestic research institutes, especially universities. GRIs have experience conducting biomedical and clinical research, but their experience conducting HPSR is limited. This gap can be partly attributed to the relatively recent emergence of HPSR as a field. A related challenge is limited human resource capacity for HPSR. Few senior researchers in the country focus on HPSR, which limits the available capacity to generate high-quality HPSR on key topics of interest to policymakers. This gap is exacerbated by the high turnover of HPSR researchers, especially at universities, driven by a lack of staff retention mechanisms, poor incentives, and generally unsupportive research environments. Taken together, these factors negatively affect research institutes’ capacity to generate high-quality evidence.

#### Inadequate HPSR funding practices

Study participants indicated that the lack of core funding for HPSR has affected institutional capacity to both generate and make use of research evidence by policymakers. Ethiopia is not the only country where this is the situation; a study in LMICs documented that lack of core funding for HPSR remains a key institutional capacity challenge [22]. Limited budgets affect institutions’ capacity to disseminate their findings and communicate about research evidence, including in policy-making platforms. Researchers reported lacking skills in preparing and managing budgets, and the institutions lacked structures to support them in these tasks. Inefficient financial management and poor record-keeping practices within research institutions have undermined the finance and procurement processes for research. Thus, in addition to providing sufficient and reliable funding to support HPSR, institutes and universities must also develop better budgeting guidelines and other project support on budget management for researchers. Unless these underlying roots are addressed, simply increasing budgets for HPSR will not result in more research, and Ethiopia would continue to experience problems with the quality and quantity of HPSR generated in the country.

The amount of research budgets and how it is being used have consequences for the relevance of the research to national policy priorities, concerns, and demands. Research institutes and the government need to establish clear research priorities and agendas, at both national and sub-national levels, to ensure that funding received is being allocated to relevant research issues transparently and efficiently [19]. Donors should also work to ensure that the money they contribute is dedicated to meeting national and sub-national research priorities that will contribute to societal development through strengthening health systems.

### Research evidence uptake and barriers

The little evidence that is generated by the HPSR institutes does not get into policies and practices due to various factors such as poor quality, lack of policy relevance (fit for purpose), ineffective dissemination and communications, and lack of evidence culture in policy communities. Consequently, the HPSR evidence generated is insufficient and ineffective to meet the policy needs of the health system and is therefore considered a waste of resources.

This study found Ethiopia’s health system has a little culture of seeking out research evidence for policy decision making, though there are some recent positive efforts such as the RAC initiative. Study respondents indicated that the majority of recent program and policy decisions were not informed by research evidence. Instead, decision-makers relied on past experiences, the opinions of experts, political considerations, and feedback provided during program management supervision and review meetings. While some respondents cited poor quality as a barrier to uptake of the available evidence, this study shows that the greatest challenge in Ethiopia is the disconnectedness of research institutes (especially universities) from policymakers and policy-making structures. This disconnect leaves a vacuum between those creating evidence and those in a position to use it for policy development. The disconnect arises from multiple factors at multiple levels. Policymakers generally perceived universities poorly, assuming that they produce evidence that is neither relevant to priority national health policy issues nor trustworthy or credible. Thus, policymakers’ mindsets are unreceptive to available evidence.

The notion that “*research is a waste of resources and has no value,*” is widespread. Such perception is not to deprive of value HPSR, rather it reflects a feeling of despair or lack of hope among participants due to the absence of tangible and promising research utilization strategies and policies embedded in decision-making processes that enforce and ensure HPSR conducted in the country are relevant to policy, timely disseminated with a transparent system of accountability to make use of it. Studies in other LMICs where linkages are missing between HPSR practitioners and policymakers have reported similar challenges [22–25]. Another challenge is that policymakers lack the capacity and expertise that would enable them to access, select and interpret available evidence. This also contributed to poor uptake of evidence in Ethiopia and has also been reported in other settings [25–28].

#### Ineffective HPSR dissemination and communication

Many researchers lack competency in timely and effective communication to stakeholders and policymakers about research results and policy recommendations that emerge from the findings; this, combined with the absence of institutional platforms to support dissemination efforts, leaves research outputs languishing on the shelf as related policies are formulated. Researchers must improve their approaches to presenting and communicating research findings [25]. On the policy makers’ side, the decision-making environment is not supportive of engaging policymakers and program managers to demand and use evidence for decision and policy development, nor are there any incentives in place to encourage them to do so. Earlier studies in LMICs also reported similar challenges [22, 24].

The engagement of both policymakers and researchers alike was identified as a key factor in bridging the gap between research and policy. Currently, there is no effective platform to promote the translation of evidence into policy in Ethiopia. However, the recent creation of one RAC as a discussion forum in MOH has already meaningfully enhanced demand for and uptake of evidence. This model needs to be replicated across programs and health system levels. Other related problems must also be addressed, including gaps in leadership and governance in HPSR, the lack of accountability, and the absence of rule of engagement for both the researchers and decision-makers. These constraints have been documented in other countries as well [24, 25, 29–35].

In this regard, Ethiopia can learn from experiences in India, Cambodia, Nigeria, and Bangladesh. These countries have improved their HPSR capacity through a combination of interventions, including strengthening the capacities of institutions and individuals (both for researchers and policymakers); and building infrastructural support and platforms to create easy access to research outputs [36]. A systematic review in the African Health system also shown that strategies such as increasing access to quality research outputs and guidelines, enhancing capacity, leadership, and political will combined with technical support, regular clear communication, and framing of evidence as policy issues, participatory engagement of research stakeholders such as joint priority settings, researcher and networking improve evidence uptake [37]. Moreover, techniques including policy briefs, capacity-building workshops, and policy dialogues, boosting local research capacities improve research evidence translations into policy and practice [37]. Thus, researchers should be prepared and supported to develop diverse approaches to communicating their findings, such as producing policy briefs and other short summaries. Finally, efficient and effective project management systems are necessary to ensure that research findings reach their intended audiences.

To the best of our knowledge, this study is the first of its kind in Ethiopia in assessing the institutional capacity among Ethiopian HPSR institutes in generating policy-relevant evidence and policymakers’ interest and experience in using evidence for decision making on health. However, the study was not without limitations. Lack of proper organizational documentation and incompleteness of data on issues such as funding management, and management of research outputs might be affected the depth of the information presented here. Although inflows of funds from different sources (internal and external) are well regulated, accurate records of these flows were hardly available.

## Conclusions

This study found that despite positive trends and improvements, the institutes conducting HPSR remain limited in number, the quality of research remains concerning, and the supply of policy-related research is insufficient. There is still too little recognition, knowledge, or awareness of HPSR in Ethiopia. Within research institutions, HPSR is not prioritized; there is little funding and support devoted to it and the institutions have inadequate human resource capacities to undertake high-quality policy-relevant research. The HPSR research that is being produced in Ethiopia is characterized by a lack of engagement with policymakers, including weak or absent links between supply and demand. Policymakers expressed major concerns that have affected the entire endeavor of conducting research to improve policy, including lack of trust in the credibility of research, the lack of timeliness and completeness of studies, and the irrelevant and unfeasible policy recommendations that emerge from the research. This lack of interest in research among policymakers and programmers has resulted in poor uptake of evidence for policy decision-making. However, without research evidence, policies are formulated based on opinion, political motivations, anecdotal experience, and global frameworks and declarations that may have little relevance on the ground. These factors are critical to consider in any policy-making process, but they should be combined with rigorously generated research evidence. Several other factors on the policymakers’ side (such as lack of accountability, no support framework or platform for interacting with HPSR producers, poor motivation, lack of capacity and expertise to make use of the available evidence also prevent research evidence uptake for policy and program design. Yet it is possible—and crucial—to bridge the gap between researchers and policymakers so that they can work together to use evidence for better policymaking. The key lesson and message obtained the present study is that Ethiopia has foundations in place to build a strong HPSR sector that can support its ongoing and ambitious health system strengthening efforts. Building on that foundation will require commitment and investment from researchers and their institutions to enhance the generation of high quality and sufficient evidence for effective policy-making. Through capacity development, institutional strengthening, and intentional relationship-building, researchers and policymakers can come together to advance their common goals. Most importantly, researchers’ engagement in the policy circle and policymakers’ engagement in process shapes policy relevant research and support policy decision.

## Data Availability

The datasets during and/or analyzed during the current study are available from the corresponding author on reasonable request.

## Abbreviations

AHRI: Armauer Hansen Research Institute
COREQ: Consolidated criteria for reporting qualitative research
EPHI: Ethiopian Public Health Institute
EBP: Evidence-Based Practice
FMOH: Federal Ministry of Health
GRIs: Government research institutes
GTPII: Growth and Transformation Plan
HPSR: Health policy and system research
HSDPs: Health sector development plans
HSEP: Health Service Extension Program
IIPHC-E: International Institute of Primary Health Care in Ethiopia
KII: Key informant interview
MCH: Maternal and Child Health
LMICs: Middle-income countries
MOSHE: Ministry of science and higher education
NCDs: Non-communicable diseases
NGOs: Non-governmentally organizations
RHBs: Regional health bureau
RAC: Research Advisory Council
UNICEF: United Nations Children’s Fund
USAID: United States Agency for International Development
WHO: World health organization
ZHOs: Zonal health office
